# Supplementary material for: Unveiling gender inequality in the US: Testing validity of a state-level measure of gender inequality and its relationship with feminist online collective action on Twitter
Source: PLoS One. 2024 Jul 17;19(7):e0306121. doi: 10.1371/journal.pone.0306121 (PMC11253932; doi:10.1371/journal.pone.0306121)
Supplement: Attachment — Submitted filename: Response to Reviewers.docx [file pone.0306121.s001.docx]

Dear Dr. Avanti Dey,

Thank you for considering our revised manuscript titled "Unveiling Gender Inequality in the US: Testing Validity of a State-Level Measure of Gender Inequality and its Relationship with Feminist Online Collective Action on Twitter" for publication in PLOS ONE. We have carefully addressed the feedback from the reviewers to improve the clarity, accuracy, and depth of our research. We believe that the manuscript has improved thanks to this round of revisions and we are grateful for the helpful comments provided to us by the editor and the reviewers.

In short, in our revision we made the following major changes:

1. We re-analyzed our data considering important factors suggested by the reviewers. This led to more precise and insightful conclusions.
2. We expanded the discussion of our findings, better connecting them to the existing literature and emphasizing their importance in understanding both gender inequality and online activism.

Below, we address your comments and each of the editor and reviewers’ comments. We hope we have adequately addressed each of the comments but if further changes are required, we would of course be happy to oblige. We hope that the changes meet PLOS ONE's high standards.

Sincerely,

Bruno Gabriel Salvador Casara, Alice Lucarini, Eric D. Knowles, and Caterina Suitner

**EDITOR**

The manuscript has been evaluated by two reviewers, and their comments are available below.

The reviewers have raised a number of major concerns. They feel the manuscript should outline a clearly-defined research question, and they request improvements to the reporting of methodological aspects of the study, for example, regarding the exclusion criteria and more information on how the data collection was completed. The reviewers also note concerns about the statistical analyses presented and request re-analyses be completed.

Could you please carefully revise the manuscript to address all comments raised?

**Response:** Thank you for forwarding the insightful comments from the reviewers. We have read and carefully considered and answered each point raised. We deeply appreciate the time and expertise the reviewers have dedicated to evaluating our manuscript, and we recognize the value their feedback brings to our work. We believe that the manuscript has improved by this round of revision, thanks to these helpful comments. Below, we address all comments, providing specific details about how we have modified the manuscript.

We hope you and the reviewers find the changes we have made to your satisfaction, and we are ready to make any further revision you consider necessary.

Sincerely,

*Bruno Gabriel Salvador Casara, Alice Lucarini, Eric D. Knowles, & Caterina Suitner*

**REVIEWER #1**

**POINT 1:** *The paper examines the validity of the Gender Inequality Index-S, a state-level measure of the GII in the US. To this aim, Study 1 tested how the GII-S correlates with some wellness measures among women and the Gini coefficient. Study 2 examined the link between GII-S and participation in the #MeToo movement by analyzing geo-localized messages on Twitter.
I enjoyed reading this paper, which is engaging, well-written, and generally clear. The issue has relevant implications, and the methodology is innovative.*

**Response:** Thank you for taking the time to review our paper and for your positive feedback, we are glad to hear that you appreciated it and that you acknowledged the issue we addressed.

**POINT 2:** *I only have some suggestions that I hope will improve the manuscript.
First, I find the title misleading. I expect the paper to focus on #MeToo; however, the analysis of #MeToo-related Twitter messages is basically functional to demonstrate the usefulness of the GII-S in predicting participation in feminist collective action and other variables. I suggest finding a more meaningful title, considering the manuscript's focus.*

**Response:** We thank the Reviewer for raising this point. We understand your concerns regarding the title's alignment with the manuscript's focus, we have now changed the title of our manuscript into:

**“Unveiling Gender Inequality in the US: testing validity of a state-level measure of Gender Inequality and its relationship with feminist online collective action on Twitter**”

We hope that this title can better emphasize the application of the GII-S in predicting participation in feminist collective action.

**POINT 3:** *On page 5 (I guess; I cannot see the page numbers), the authors should provide references for this sentence: "While massively popular, the movement has encountered a great deal of protest and criticism, especially among those who endorse a more traditional worldview regarding gender roles." Whereas I can see the point here, the following sentences refer to literature published before the #MeToo phenomenon. However, there are studies that, for instance, related sexism and attitudes toward #MeToo. Even though such studies focused on attitudes or intentions to engage rather than actual participation, they would provide some context for the sentence mentioned above (e.g., Kunst et al., 2019; Menegatti et al., 2023; Moscatelli et al., 2021)*

**Response:** Thank you for this useful feedback, we have now integrated into our introduction these references, to properly contextualize the sentence on the reception of the #MeToo movement. These changes can be found at p. 3 of the revised version of our manuscript and are reported below:

“Indeed, studies indicate that sexist beliefs and perceptions of #MeToo's effects significantly affect individuals' willingness to support the movement [28]. Moreover, Kunst and colleagues [29] reported that men's negative perceptions of #MeToo were linked to higher levels of hostile sexism and lower feminist identification, highlighting ideological rather than gender-based divisions. Additionally, internalized sexualization among women in Italy was found to negatively affects attitudes toward #MeToo and suggests that such internalization may reinforce ideologies that hinder the movement's goals [30].”

**POINT 4:** *I would avoid reporting a summary of the current research’s main results at the end of the introduction (page 6).*

**Response:** Following your suggestions, we have now removed the summary of main results from the introduction. The revised –and briefer– version of this paragraph can be found at p. 4 of the revised version of our manuscript and is reported below:

“Across two studies, the present research employs the Gender Inequality Index (GII) and establishes its utility in assessing state-level differences in gender disparities within the US.

Study 1 employed population-based data retrieved from several platforms for big data storage, to create and test the validity of the state-level Gender Inequality Index (GII-S), namely an adaptation of the GII allowing within-country comparisons, which assesses state-level gender inequality across US states. In Study 2, we employed Twitter-based data to validate the GII-S externally, by examining its correlation with the prevalence of #MeToo tweets as a marker of feminist collective action and to explore the interplay between gender inequality, political ideology, and such collective actions across states.”

**POINT 5:** *The description of the GII reported in the Measures selection (pages 6-7, before the section on Calculation of GII-S scores) should better be moved to the theoretical introduction.*

**Response:** In line with the Reviewer’s suggestion, we have now moved this section at the end of the Introduction (p. 4 of the revised version of our manuscript)

**POINT 6:** *How did the authors choose that specific period for the collection of Tweets? I suggest they say something about their choice.*

**Response:** Thank you for this suggestion. We have decided to consider the timeframe from mid-January to mid-February 2019 as it aligns with the peak period of activity of Tweets on the #MeToo movement, according to an analysis of Google Trends data spanning the past five years. We have now outlined the rationale behind our decision, this information can be found at p. 8 of the revised version of our manuscript and is reported below:

“This specific timeframe was selected as matches the peak period of activity for the movement, based on Google Trends data, over the past five years (Fig 3), capturing the height of public engagement and discourse on the Google platform.”

**POINT 7:** *Concerning Study 2, the finding that gender inequality is associated with lower MeToo participation is interesting and led me to wonder whether there could be other variables that are not taken into account here. Could it be, for instance, that women’s age plays a role? Could it be that states with higher gender inequality also have an older female population?*

**Response:** Thank you to this very useful comment that helped us to reflect more deeply about potential confounder variables. We ran a further analysis checking whether taking into account women median age by states in 2019 would suppress the effect of GII scores on #MeToo posts frequency. While GII scores were still significantly associated with #MeToo posts frequency (*r* = -.46, *p* = .001), no evidence for an association with median women age was found (*r* = -.19, *p* = .22). Moreover, we checked whether the association between GII and #MeToo frequency was robust also when economic inequality and GDP per capita were taken into account. Once again, GII scores were significantly associated with #Metoo frequency (*r* = -.45, *p* = .003).

We have now updated the results of Study 2, in order to include this more severe test of the GII-S –#Metoo frequency association. Importantly, Importantly, we have now updated the Statistical Analyses section (p. X of the revised version of our manuscript), in order include this more severe test of the GII-S – #MeToo frequency association. (p. 9 of the revised version of our manuscript):

“Furthermore, we employed the Bayes Factor, a statistical measure used to evaluate the strength of evidence in favor of one statistical model over another, to test different models to compare the predictive strength of GII-S scores against other models. Specifically, we calculated Bayes factors, using the software JASP [49], for three linear models using #MeToo tweet frequency as dependent variable. The first (null) model included the state-level frequency of general tweets, which should trivially predict the use of any given hashtag (including #MeToo), state-level median women age, GINI, and GDP per capita. In the second (GII) model, we added state-level GII as a predictor of #MeToo tweet frequency. Finally, in the third (political) model, we added state-level political orientation, instead of GII, as a predictor of #MeToo tweets.”

Consistently, we have also updated results, in the appropriate section of the revised version of our manuscript (p. 9):

*“*According to the Bayes Factors analysis, the GII model was superior to both the null and political models. In particular, the observed data pattern is approximately twenty-eight times more likely to occur under GII model than null model, which can be interpreted as strong evidence [50] in favor of the model with GII as predictor.”

**POINT 8:** *Finally, the authors should devote more effort to discussing their research’s theoretical and practical implications.*

**Response:** Thank you for your suggestion, we have now completely reframed the discussion taking into account also the suggestions from Reviewer 2. All results from Study 1 and 2 are now more deeply discussed, and theoretical interpretation are given for all our findings. Moreover, we added a paragraph at the end of the Conclusions section, where some practical implications are more clearly outlined (p. 14):

“(…) the practical implications of these studies are extensive in several respects. First, by confirming the reliability of the GII-S, policymakers and advocates can use an effective tool to examine and tackle gender disparities in specific areas of the country.

Second, the outcomes of Study 1 emphasize the need to address gender inequality not just as a social justice issue but also as a means to boost in particular women’s but also men’s overall well-being. The awareness of the negative correlates of gender inequality for everybody can be an important element in gathering support from a larger part of the population.

Finally, Study 2 offers important insights into how gender inequality impacts collective action. The inverse relationship between GII-S scores and involvement in the #MeToo movement indicates that greater gender inequality may reduce awareness and activism regarding sexual harassment and violence against women. This is particular, relevant for activists and organizations interested in promoting gender equality, as they can consider that stronger efforts are required to mobilize people in contexts that would likely benefit most for interventions.”

**REVIEWER #2**

**POINT 1:** *Thank you for the opportunity in review this interesting work. The paper is well-written and relevant to current literature but has one important limitation not sufficiently mentioned. The structure of the paper also seems a little strange. Find below some suggestions to improve the clarity and reading of the work.
Title: The approach of use tweets should be highlight in the title. Readers could think this is a population-based study, but conclusions are based on a very specific sample.*

**Response:** We sincerely appreciate the time you invested in reviewing our paper and we thank you for recognizing the significance of the issue we addressed.
Concerning the title, we align with your comment and we acknowledge that, in its current form, the title might be misleading, a point that was also raised by Reviewer 1. We have now changed the title, and we hope it is now in line with both your feedback and that of Reviewer 1. As reported above, the new title is:

**“Unveiling Gender Inequality in the US: testing validity of a state-level measure of Gender Inequality and its relationship with feminist online collective action on Twitter**”

With this changes, we have tried to better outline the online context of our work, by mentioning Twitter in the title. We did, however, chose not to directly emphasize the use of Twitter-based data in the title because, despite Study 2 is not a population-based study (being based on Tweets frequency), Study 1 is. Indeed, to calculate GII-S scores we relied on data from several platforms for big data storage, such as Gallup and the United State Census Bureau, whose data are population-based. Currently, the new title of our manuscript does meet your suggestion to underline the online component of this work, as we refer to online feminist collective action on Twitter, while also aligning with Reviewer’s 1 comment, who suggested to put more focus on the GII-S and on its predictive role of feminist collective action. We hope that this solution aligns with your request.  **POINT 2:** *Introduction: The text is well-written and present relevant arguments, however, is too long.
I suggest remove the 4th paragraph that is currently not adding too much.*

**Response:** In line with your suggestions, we have now removed the 4th paragraph from the revised version of our manuscript.

**POINT 3:** *The three paragraphs about GII could be only one with 4 points briefly: a) why measure GI; b) GII is the most used instrument; c) limitations of GII; d) between countries issues.
The paragraphs about USA are too long too. I suggest focus on Mee too directly e reduce the text drastically (only one paragraph about USA/Me too). All these statements could return in the Discussion section.*

**Response:** Thank you for your constructive feedback. In response to your comments, we have streamlined the contents discussed in the Introduction, to ensure it remains concise and focused on the core aspects of the Gender Inequality Index (GII) and the #MeToo movement's impact in the USA. Here's the revised version that incorporates your suggestions (p. 2):

“Fighting gender inequality requires measuring it, and specific instruments have been developed to assess disparities between men and women. One of the most important measures of gender inequality is the Gender Inequality Index (GII; [26]), a measure developed to assess gender inequality at country level, but never used for within-country comparisons. Although country-level comparisons are relevant for providing a broader perspective on social phenomena across a range of cultures and contexts, they often face challenges due to varying data collection methods and cultural differences, introducing methodological inconsistencies (e.g., “qualitative” ideological differences, [27]) and making strict quantitative analysis difficult. Conversely, within-country comparisons, such as between regions within the same nation, can offer more reliable insights into structural phenomena such as gender inequality, by avoiding these issues, benefiting from a unified political system and standardized data practices.”

“The United States offers a unique setting for comparing gender inequality within a country. Despite shared cultural elements and uniform data collection by federal agencies, US states exhibit significant diversity in demographics, laws, and attitudes on issues linked to gender inequality themes. Importantly, a within-country comparison across US states also provides an opportunity to focus on a specific feminist movement that was born in the country and rapidly became one of the biggest inspirations for collective action in the last decade—namely, the #MeToo movement.

While massively popular, the movement has encountered a great deal of protest and criticism, especially among those who endorse a more traditional worldview regarding gender roles.”

**POINT 4:** *Methods: The Methods section is a little confuse. My suggestion is the following:
a) Study 1 and 2 explanations⎝ what they are, and which data is used with which aims (remove hypothesis).
For example: Study 1 aims proposes a GII calculates at state level and study 2 test the external validity. The sample used for study 2 was (tweets collection) …
Ethics…
b) Remove all part about limitations of GII because it is already mentioned in the introduction.
c) Calculation of GIIS scores ⎝ maintain topic
d) Convergent and Discriminant Validity Measures⎝ maintain topic, except Table 1 description (Table 1 is results from study 1)
e) Statistical analysis ⎝ explain the description and Pearson correlation should be used. Why were these statistical methods used? Why you think correlation is a good metric for test your hypothesis?
f) Adjustments and models also should be explained in the Methods section – analysis subsection.*

*Results: Table 1 should be described in the Results section.
In results section, please explain what id “Hx was supported’. Reader needs to red Methods many times to remember what each Hypothesis is. Please re-write this section.*

**Response:** Thank you for this comment, we have done our best to restructure the method section following your suggestions. In particular,

a) We have added a short paragraph named “The present research”, which provides to the reader an overview of our studies and their aims, also mentioning the samples employed, in line with your request. Consistently, we dropped detailed hypotheses from the Method section, exclusively focusing on the study aims and the data used.

b) We have removed the discussion on the limitations of the GII from the Methods section, as these were already addressed in the introduction.

c/d) Under the method section of Study 1, we have added two sub-paragraphs: one specifically focusing on the calculation of GII-S scores and one testing its convergent and discriminant validity. This, in line with the Reviewer’s suggestion, could ease the readers, so that they can properly distinguish the core steps of the development and validation of the GII-S.

e/f) For both Study 1 and 2, we have added a short “Statistical Analysis” paragraph, in which we outline the analyses employed and the rationale behind our decisions. Importantly, the choice of Pearson correlation (Study 1, Study 2) and Bayes Factor (Study 2) has been now explicitly justified by their relevance to testing our research questions. Adjustment and models are now explicitly formalized in the statistical analysis section.

g) In line with you request, results from Study 1 and Table 1 have been moved in the results section. Additionally, we removed references to the hypotheses (e.g., “Hx was supported” sentences), as hypotheses are no longer formalized.

**POINT 5:**  *Discussion: Plos’s papers have only one Discussion section. Please, group the discussion regarding studies 1 and 2 in the general Discussion.* *Topic removed from introduction could return in this section.*

**Response:** In line with the Journal’s standards and with the Reviewer’s suggestion, we have now created a new section named “General discussion” that integrates information from the –previously separated– discussion sections of Study 1 and 2, which have been deleted. Importantly, following the Reviewer’s suggestion, we moved the discussion about the USA context, that we previously reported in the introduction, in the general discussion.

**POINT 6:** *The paper has one big limitation that is not mentioned. Data from study 2 are not based on a population-based study but in tweets, a very biased sample. Who are the people using internet in USA? Additionally, who are the people with active voice in the USA?*

*For example, what is the opinion about GI or ‘Me too’ movement in very poor population, far from internet access? Feminism and women rights could be not even a priority for a family without milk or bread for the meals.*

*I believe, this should be strongly reinforced as a limitation and believe all conclusions should have caution in interpretation.*

**Response:** Thank you with this useful comment. We agree with you that it is important to consider the opportunity for internet access. Coherently, we tested the association between #MeToo tweets and GII-S scores taking into account the general frequency of messages on Twitter by state. This variable is a proxy of the use of Twitter by state: the more the proportion of general tweets in a State the more Twitter is used and it is likely that more people have in general more access to it. At the same time, we agree that economic factors can be relevant in this context and therefore we updated our analyses by testing the GII-S association with #MeToo tweets by taking into account two important economic factors at the state level, namely economic inequality, measured with GINI, and State-level wealth, measured with GDP per capita. Our analysis reveals that economic factors like GINI and GDP per capita do not diminish the association between the Gender Inequality Index (GII) and the frequency of #MeToo tweets. This finding suggests, gender inequality, as captured through the lens of the #MeToo movement, transcends economic disparities at the State level. It indicates that discussions surrounding feminism and women's rights, as highlighted by the #MeToo movement, are prevalent and resonate across states that vary in terms of economic inequality and prosperity. A limitation that cannot be addressed by this research is that our results are about States and not individuals, therefore they cannot be generalized to individuals. We now mention this limitation in the general discussion section (p. 14 of the revised version of our manuscript):

"Another important limitation of our analysis lies in the fact that it is based on aggregated state-level data, which limits our ability to extrapolate our findings to individuals directly. Although our study identified the Gender Inequality Index at the state level (GII-S) as a significant predictor of participation in the #MeToo movement, even when taking general use of Twitter and state-level economic performances into account, it remains unclear whether individuals that not have the opportunity to use Internet (and Twitter) and people with lower socio-economic status have a stronger or a weaker motivation to engage in feminist collective actions. Indeed, in our studies we do not provide information about the perspectives of individuals. Consequently, survey studies that focus on individual-level analysis are essential to determine if personal socio-demographic characteristics influence attitudes toward feminist online collective actions, such as #MeToo"
