# Supplementary material for: Unveiling gender inequality in the US: Testing validity of a state-level measure of gender inequality and its relationship with feminist online collective action on Twitter
Source: PLoS One. 2024 Jul 17;19(7):e0306121. doi: 10.1371/journal.pone.0306121 (PMC11253932; doi:10.1371/journal.pone.0306121)
Supplement: Attachment — Submitted filename: Response to the reviewer.docx [file pone.0306121.s002.docx]

**EDITOR**

Dear Dr. Salvador Casara,

Thank you for submitting your manuscript to PLOS ONE. After careful consideration, we feel that it has merit but does not fully meet PLOS ONE’s publication criteria as it currently stands. Therefore, we invite you to submit a revised version of the manuscript that addresses the (minor) points raised during the review process.

 Due to a change in the Editor, I, as a guest Editor, asked the opinion of a third reviewer. The reviewer is positive about the manuscript and provided important suggestions, which I invite you to follow.

**Response:** Thank you for forwarding the insightful comments from the reviewer. We have thoroughly reviewed and carefully addressed each point raised. We sincerely appreciate the time and expertise the reviewer has dedicated to evaluating our manuscript, and we acknowledge the significant value their feedback adds to our work.

As a result of this revision, we believe the manuscript has improved considerably, thanks to these constructive comments. Below, we provide a detailed response to all comments, explaining the specific changes we have made to the manuscript.

We hope that you and the reviewer are satisfied with the revisions we have implemented. We remain open to making any additional revisions you may deem necessary.

Sincerely,

*Bruno Gabriel Salvador Casara, Alice Lucarini, Eric D. Knowles, & Caterina Suitner*

**Reviewer**

*Thank you for the opportunity to revise the manuscript entitled “Unveiling Gender Inequality in the US: testing validity of a state-level measure of Gender Inequality and its relationship with feminist online collective action on Twitter”. This is a revised version of the manuscript and I think that the authors adequately addressed the comments raised by the Reviewers. The results of the two studies contribute to the understanding of gender inequality and its social and psychological correlates in the US, providing useful insights for practical implications toward greater equality. The comments that follow are minor and they point to the overall improvement of the manuscript.*

**Response:** We are pleased to hear that you believe we have adequately addressed the comments raised by the Reviewer, and we thank you for your constructive feedback.

**POINT 1:** *I suggest revisiting the occasional use of casual language, whether implicit or explicit, given the adopted correlational design. Precision is critical in eliminating any potential for misunderstanding.*

**Response:** Thank you for your valuable feedback. We have carefully reviewed the manuscript and identified instances where language could be interpreted as implying causality. These instances have been revised to ensure clarity and precision, reflecting the correlational nature of our study. We have replaced any language that may have suggested causal relationships with terminology that accurately describes associations and correlations. For example, we are now avoiding the term “predicted” and we are using the term “associated”.

**POINT 2:** *My second comment regards the lack of significant association between the GII-S and GINI coefficient. The authors discuss that “economic and gender disparities represent distinct aspects of social inequality”. However, there is literature suggesting that economic and gender inequality are related to each other (see Moreno-Bella et al., 2023 for a recent paper). Usually, societies characterized by higher levels of economic inequality are also characterized by higher levels of gender economic inequality (i.e., gender pay gap). In economically unequal societies, women are usually disadvantaged compared to men. Therefore, I would suggest being more cautious in interpreting this lack of significant association. This could be due to the specific context the authors focused on (i.e., the US) or the specific measure they used (i.e., if I understood correctly, the GII-S does not account for gender economic inequality).*

**Response**: Thank you for your valuable feedback. We have carefully considered your comment regarding the lack of significant association between the GII and the Gini coefficient. While we see the consistency of our result with other empirical evidence showing that the relationship between GII and GINI is not necessarily positive nor negative, we completely agree with the point raised by the reviewer. Thus, we added the following paragraph (p. 12):

“Our findings indicate that GII-S scores are not significantly correlated with economic inequality, as measured by the state-level Gini coefficient. Our findings align with the idea that economic inequality and gender inequality are distinct phenomena. For example, reducing disparities between men and women does not automatically imply that economic differences between classes will follow the same trend. Moreover, coherently with our results, from the 1990 Country-level gender inequality measured with the GII is decreasing in the World and in many countries, USA included [62]. Differently, economic inequality appears to be more stable over time and it increased in the USA during the same time period [63]. However, it is important to notice that there are social issues representing both facets of gender and economic inequality, such as the gender wage gap. Furthermore, our data shows that the Gini coefficient is associated with women’s financial well-being (but not men’s), and there is previous research reporting a link between economic and gender inequality [64]. Additionally, the distinctiveness between the two issues may be particularly salient because of the specific aspects of gender inequality that the GII-S measures, which do not directly account for gender economic inequality. Therefore, we advise caution in interpreting the lack of a significant association between these two measures.”

**POINT 3**. *Along the same lines as my previous comment, the authors discuss the negative association between the GII-S and MeToo hashtags from the perspective of the system justification theory. This could be a possibility, but further evidence is needed before concluding that e.g., GII-S undermines feminist collective action through higher perceptions of gender inequality as legitimate. Again, the authors should be more cautious in interpreting these results and propose additional explanations. For example, given the specific inequality dimensions accounted by the GII-S, could be that this negative association is due to the lack of resources, empowerment, and group efficacy beliefs? Relatedly, this could be due to the specific measure the authors used for collective action (i.e., MeToo hashtags) and other measures of collective action engagement (i.e., demonstrations, strikes) should be examined.*

**Response**: Thank you for your comment which helps us to provide multiple interpretations for our results. Now, we are suggesting that our results are coherent with the SJT account, but we also consider other theoretical perspectives coherent with our results. We add this in the discussion section (p. 13):

“Results showed that states with high levels of gender inequality tended to produce fewer #MeToo tweets than did states low in gender inequality. One possible interpretation of these findings can be framed in the theoretical account of the System Justification Theory, in line with our preregistered hypothesis (SJT; [65]). According to SJT, people in macro-level contexts characterized by a long history of patriarchy and adherence to traditional sex roles will be motivated to justify gender inequality—a process that likely reduces interest in feminist collective action. In this way, inequality’s very existence may further resistance to change. However, it is important to highlight that, while our results are consistent with an interpretation based on System Justification Theory (SJT), they do not conclusively prove it. Other interpretations are also plausible. For instance, according to the Social Identity Model of System Attitudes (SIMSA; [66]), the association between higher GII scores and lower frequency of #MeToo hashtags might be due to accuracy motives. These motives could lead individuals to passively perceive and acknowledge the status quo without actively defending or maintaining the system. Additionally, as the GII measures objective structural inequalities between men and women, it is possible that in States with higher levels of gender inequality, there is a lack of resources and opportunities to participate in the #MeToo movement. Resource scarcity may also represent an obstacle in the expression of important psychological antecedents of collective action, such as feelings of empowerment and group efficacy beliefs [67]. It is also worth considering that our measure of collective action engagement was specific to the #MeToo hashtags. Future research should examine other forms of collective action, such as participation in demonstrations and strikes. By doing so, researchers might gain a more comprehensive understanding of the dynamics at play, while also providing a more direct test of their possible explanation.”
